# Effectiveness of tailored digital health interventions for mental health at the workplace: A systematic review of randomised controlled trials

**DOI:** 10.1192/j.eurpsy.2023.1810

**Published:** 2023-07-19

**Authors:** C. M. van der Feltz-Cornelis, T. M. Byrne, J. Shepherd, D. Merecz-Kot, M. Sinokki, P. Naumanen, L. Hakkaart-van Roijen, C. Empower

**Affiliations:** 1 Health Sciences; 2 HYMS, University of York, York, United Kingdom; 3 Institute of Psychology, University of Lodz, Lodz, Poland; 4 Turku Centre for Occupational Health, University of Turku, Turku, Finland; 5 Erasmus School of Health Policy and Management (ESHPM), Erasmus University Rotterdam, Rotterdam, Netherlands

## Abstract

**Introduction:**

Mental health problems in the workplace are common and have a considerable impact on employee wellbeing and productivity. Mental ill-health costs employers between £33 billion and £42 billion a year. According to a 2020 HSE report, roughly 2,440 per 100,000 workers in the UK were affected by work-related stress, depression, or anxiety, resulting in an estimated 17.9 million working days lost.

This study is part of the EMPOWER study. The European Intervention to Promote Wellbeing and Health in the Workplace (EMPOWER) consortium’s aim is to create an individualised digital tool that promotes employee wellbeing, mental health, and work productivity. It has received funding from the European Union’s Horizon 2020 research https://ec.europa.eu/programmes/horizon2020/en/home) and innovation program under grant agreement No 848180.

**Objectives:**

We performed a systematic review of randomised controlled trials (RCTs) to assess the effect of tailored digital health interventions provided in the workplace aiming to improve mental health, presenteeism and absenteeism of employees.

**Methods:**

We searched several databases for RCTs published from 2000 onwards. Data were extracted into a standardised data extraction form. The quality of the included studies was assessed using the Cochrane Risk of Bias tool. Due to the heterogeneity of outcome measures, narrative synthesis was used to summarise the findings.

**Results:**

Seven RCTs (eight publications) were included that evaluated tailored digital interventions versus waiting list control or usual care to improve physical and mental health outcomes and work productivity.

The results are promising to the advantage of tailored digital interventions regarding presenteeism, sleep, stress levels, and physical symptoms related to somatisation.

There is less evidence for addressing depression, anxiety, and absenteeism in the general working population, but they significantly reduced depression and anxiety in employees with higher levels of psychological distress.

**Conclusions:**

Tailored digital interventions seem more effective in employees with higher levels of distress, presenteeism or absenteeism than in the general working population. However, so far, there are not many studies in this domain. Given the promising results, tailoring of digital interventions based upon employee input should be a focus in future research.

**Disclosure of Interest:**

None Declared

